# Instant Feedback Rapid Prototyping for GPU-Accelerated Computation, Manipulation, and Visualization of Multidimensional Data

**DOI:** 10.1155/2018/2046269

**Published:** 2018-06-03

**Authors:** Maximilian Malek, Christoph W. Sensen

**Affiliations:** Institute of Computational Biotechnology, Graz University of Technology, Graz, Austria

## Abstract

**Objective:**

We have created an open-source application and framework for rapid GPU-accelerated prototyping, targeting image analysis, including volumetric images such as CT or MRI data.

**Methods:**

A visual graph editor enables the design of processing pipelines without programming. Run-time compiled compute shaders enable prototyping of complex operations in a matter of minutes.

**Results:**

GPU-acceleration increases processing the speed by at least an order of magnitude when compared to traditional multithreaded CPU-based implementations, while offering the flexibility of scripted implementations.

**Conclusion:**

Our framework enables real-time, intuition-guided accelerated algorithm and method development, supported by built-in scriptable visualization.

**Significance:**

This is, to our knowledge, the first tool for medical data analysis that provides both high performance and rapid prototyping. As such, it has the potential to act as a force multiplier for further research, enabling handling of high-resolution datasets while providing quasi-instant feedback and visualization of results.

## 1. Introduction

As datasets grow ever larger, so does the importance of efficient processing by fully utilizing the information they contain. However, most data processing is still done on generic CPUs, even though programmable GPUs, capable of performing arbitrary computations, have been available on the consumer market since 2006.

Often, processing of many data types, for example, image data, is followed by some form of visualization. Most existing tools for medical images are either viewers or are focused on the processing of data. Of the viewers, there is either focus on medical insight [1], or pleasing visual rendering [2], or both, depending on the use case. Visualization of data and human intuition together can provide crucial insights into a given dataset. One example is the visualization of metadata created from patient data (e.g., 3D-renditions of data derived from x-ray images, MR scans, and CT scans), allowing patients to better understand the nature of their condition. Up to now, viewers are typically not user-programmable and only provide a limited set of parameters to adjust their output.

Twenty years ago, data transformation was integrated into the rendering process, as a direct transformation alone was too costly for the hardware available at the time [3]. The current hardware has much higher capabilities and processing of datasets, which were previously considered as too large, has become common. With the advent of programmable GPUs in the mid-2000s, GPUs are now used to perform general-purpose computations (GPGPU) to process data in parallel, reducing the computational time drastically [4–7]. Although originally designed for graphics applications, the massively parallel design of GPUs allows data processing much more efficiently than is typically possible with traditional CPUs, while at the same time reducing the hardware footprint to the size of a graphics card. Given the increased computational capabilities, using the GPU for both processing and rendering is faster and more flexible compared to earlier approaches on CPUs or fixed-function GPUs [8, 9]. GPU-accelerated computation is, however, not as ubiquitous as it could be, as the development of parallel algorithms can be prohibitively difficult, especially for those not familiar with the different programming model [10]. Initial use of GPGPU techniques involved interpreting data as textures and then performing typical graphics operations on them, such as blending, projection, or interpolation [11, 12].

As GPGPU techniques matured, dedicated libraries for GPU programming became common, of which the most well-known examples are CUDA [10] and OpenCL [13]. CUDA requires a separate compilation step and is thus inadequate for rapid prototyping. In contrast, OpenGL [14], which is typically used as a backend for graphics and rendering, has had support for a run-time compiled shading language (GLSL [15, 16]) for a long time. This means that the GLSL source code is passed to the graphics driver, which then dynamically compiles an appropriate binary representation for the platform. OpenGL 4.3, released in 2014, was extended to support compute shaders, which facilitate arbitrary computation on the graphics card directly.

We exploit this dynamism to enable interactive development in GPU-accelerated computing and data exploration, with medical image processing in mind. Specifically, we want to be able to see results immediately, even while editing the source code. This concept is used today, for example, for entertainment in the so-called Demoscene [17–19]. We expect this approach to also enable intuition-driven development in the domain of medical image analysis.

## 2. Methods

We have created a computational framework, which facilitates scriptable, rapid prototyping friendly GPU-accelerated computing and rendering of medical data. On the highest level of the user interface is a graph editor to control the underlying graph-based processing pipeline, where nodes perform operations on data, and edges between them indicate data flow (Figure 7). Internally, the framework consists of a scene graph, describing hierarchies of objects. This is in principle the same architecture skeleton as used by real-time or game engines [20]. The architecture supports creating multiple scenes, performing rendering, and subsequently either the display of the result or further processing. As an example, a volume (3D) texture can be rendered from different perspectives via the integrated volume renderer into a number of 2D textures. Since data processing happens via a flexible user-controlled data pipeline, the resulting textures can be further processed.

Key features are very rapid prototyping and short iteration times, which allow to obtain results quickly. Since large parts of the software are scripted, and all of the scripts can be changed and reloaded at any time, many features, including the user interface (UI), can be changed without restarting the framework. GPU computation is realized with compute shaders, which can be changed and reloaded in the same way as the scripts. Input/output file formats are automatically detected and support for new formats can be added via plugins. Multiple windows and screens are supported to maximize the usable space. This feature also provides support for more advanced display configurations, such as two-projector stereoscopic 3D setups or CAVEs [21].

**Figure 1:**
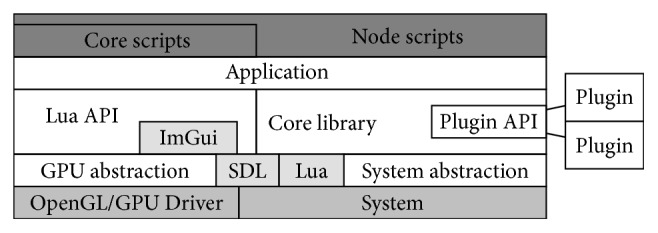
Block diagram of the software. SDL provides most of the platform-dependent functionality; everything except the lowest software layer is completely platform-independent.

Scripting is realized with Lua [22], driving application and pipeline logic, UI, and node functionality. The rest of the application and library is implemented in C++. Aside from Lua, the external libraries utilized include SDL [23] (for cross-platform support), OpenGL, and* Dear ImGui* [24] (for the UI). A custom plugin interface is implemented to enable support for extensions, third-party file formats, and additional Lua functions. The GPU backend used is currently OpenGL version 4.5 [25]. OpenGL is a cross-platform graphics API, which combines rendering and GPU-accelerated computation. The implementation of the backend follows modern AZDO (approaching zero driver overhead [26]) principles, where applicable. Accelerated processing is performed via compute shaders, which are written in GLSL. Due to the pipelined nature of OpenGL, most of its operations are performed in the background, while the main CPU can perform other tasks [25]. The core library also supports semi-automatic multithreading of CPU-bound tasks, but since the heavy compute jobs are usually performed by the GPU; multithreading is rarely necessary.  Figure 1 shows an overview of the overall framework design.

From a developer's perspective, the following functions are provided: Vertex and fragment shaders facilitate the rendering tasks and compute shaders perform the computational tasks. Shader introspection is used to determine inputs, outputs, and parameters of the particular shaders. This information is used by the UI. Textures are used for image and data storage and can be one- to three-dimensional, with 1–4 channels, using various internal formats (e.g., 8 bits to save space, 16 bits for high detail, and float for HDR data). Texture fetches can optionally be customized with a swizzle mask; that is, the order in which color components are sampled is user-controllable. GPU Buffer objects provide support for arbitrary, unformatted memory, using persistent and coherent memory mapping. Manipulating them from either, the CPU or GPU side, respectively, is possible without special constraints. The following OpenGL extensions are automatically used when supported by the system:* ARB_bindless_texture*,* ARB_gpu_shader_int64*,* ARB_gpu_shader5*, memory info, and various robustness extensions to recover from driver crashes:* ARB_robustness*,* ARB_robust_buffer_access_behavior,* and* ARB_create_context_robustness* as provided by SDL. Despite their benefits and simplicity, OpenGL compute shaders are not intended for very large datasets, as their running time is usually limited by the graphics driver; two seconds is the default for recent NVidia drivers on Windows. If a compute shader has not finished within that time limit, the shader is forcefully terminated. On Windows, the graphics driver is reset, which usually causes program termination. In order to overcome this limitation, large datasets are automatically split into smaller tiles, which are then processed individually across multiple shader invocations.

A simple custom file format to store up to three-dimensional image data is included. It supports lossless compression via the ZStandard algorithm [27] and is optimized for fast loading and simplicity in order to keep the core clean. Two optional standard plugins are provided. The first one uses ITK [28] to add support for the Bitmap, JPEG, GDCM, DICOM, GIPL, MetaImage, Nrrd, TIFF, PNG, Stimulate, VTK, Nifti, Gipl, and HDF5 [29] file formats. A full list can be found in the ITK wiki [30]. The second one uses the* stb_image* library [31] and adds support for PNG, Bitmap, TGA, HDR, JPEG, PSD (Photoshop), PNM/PPM, and GIF files. The two plugins are independent of each other, and we consider especially the latter as a good starting point for users trying to implement their own plugins. The API does not expose implementation details or the GPU backend and is version-compatible in both directions. Details about the plugin API can be found in the supplement. In order to facilitate scripting and script debugging, a built-in real-time data inspector is included, which can be used to traverse any Lua object along with attached variables, functions, and classes, making it possible to preview values and data objects where supported.

From a user's perspective, a number of nodes are already included, implementing the following filters/algorithms: curvature, derivative (edge detection), distance transform, various simple math operations (element-wise addition, subtraction, multiplication, division, power function; i.e. all functions supported by GLSL), min/max/average region filter, median filter, surface normal extraction, 3D → 2D slice extraction, convolution (Gaussian blur), type conversion, and thresholding. Two nodes accept a custom GLSL code snippet from the user, enabling live programming. The first compiles the entered code to a compute shader to process or generate arbitrary data. The second node compiles to a fragment shader that generates an arbitrary 2D texture, with limited compatibility to Shadertoy [17]. Other nodes that do not perform computation can act as data sources or sinks, although there is no clear separation between the two roles. Image or memory buffer loaders are pure sources. A volume renderer essentially transforms a 3D dataset into a 2D image given a perspective. A universal memory viewer with included hex-editor (that works on CPU and GPU memory) is useful to diagnose low-level memory layout problems.

The layered architecture allows even novice users to design a processing pipeline visually and interact with the provided widgets without any programming being required from the user. For quick familiarization with the UI, context-sensitive help, descriptions, and tooltips, as well as a general guide, are displayed when appropriate. The novice user is only limited by their knowledge of what existing algorithms do, how to use them, and how to combine them to perform a higher-level task. More advanced users can quickly develop new nodes, using GLSL for the computation and Lua for the interface; thus computation is always GPU-accelerated, and scripting facilitates the rapid development. This also enables users to quickly write prototype code for specific use cases. A node is implemented as a single Lua script, optionally containing GLSL code.  Figure 5 shows a complete example. The supplement contains more information and examples detailing the node API and implementation of custom nodes.

## 3. Results

### 3.1. User Interface

Our UI is designed for fast iteration, real-time interaction, and parameter adjustment to provide as much visual feedback as possible and to clearly highlight user errors, when (and also how) they occur. Data manipulation effectively happens by linking nodes together to form a directed acyclic graph (DAG). Color-coded connectors prevent accidental, type-incompatible connections. It is also not possible to construct cycles. Any attempt to perform these erroneous graph constructions will lead to an immediate display of an appropriate error message (Figure 6).

For developers writing their own nodes there are automatisms in place that attempt to predict input/output properties and how to invoke a compute shader for commonly used scenarios, simplifying the development even more.  Figure 5 shows an example and the software documentation provides an even more detailed explanation. If more customization is required, almost all functionality of a node can be specially implemented, including the UI.

### 3.2. Comparison to Existing Tools

We have compared our software package to MeVisLab [32], DeVIDE [33], GRAPE [34], GraphMIC [35], and FAST [4]. While FAST is a stand-alone library for OpenCL-accelerated image manipulation and visualization, the other packages are mainly focused on graph-based image processing and all of these utilize ITK, VTK [36], MITK [37, 38], or a variation of these libraries to perform the computation and rendering tasks. Consequently, they share ITK's main weakness, that is, the CPU-bound processing without (or with very limited) GPU acceleration [39]. All tools except MeVisLab are provided as open source packages.

In comparison to our package,* DeVIDE* requires detailed knowledge of ITK, as it directly maps ITK functions to graph nodes. A version of the package that does not require ITK exists but has limited functionality. The DeVIDE UI is not usable intuitively, as nodes use internal names and their function is not always clear. Connectors are neither labelled nor color-coded, and any input can be connected to any output. Mismatched connectors or cycles cause an error when trying to execute the graph. Missing parameters for a node are not signaled until an attempt is made to execute the graph, upon which an error is shown. We were unable to do anything meaningful with DeVIDE since we were unable to identify a valid combination of nodes which, when connected together, would produce output and not cause graph execution to fail. The UI has no apparent preview functionality available. DeVIDE is scriptable in Python.

*MeVisLab* is a large, well-established, commercial software package intended for rapid prototyping and image manipulation applications. It is rapid prototyping friendly in the sense that results can be quickly obtained and previews at every stage provide visual feedback. However, implementing any custom extension requires the MeVisLab SDK and a C++ compiler; therefore the actual development of custom extensions is not rapid prototyping friendly. Constructing a graph containing a cycle resulted in a crash. MeVisLab is scriptable in Python.

*GraphMIC* is only available for macOS; thus we decided not to test it, as we wanted to focus on platform-independent packages. From the documentation, it seems to be very similar to DeVIDE concept-wise, but it should be more rapid prototyping and user-friendly, since it supports previews, parameters can be adjusted directly on the nodes, and input/output connectors are clearly labelled. This package is also scriptable in Python.

*GRAPE* is similar to GraphMIC and DeVIDE and mentioned for completeness.

*FAST* is not a graph editor, but a C++ library similar to ITK and VTK that aims to cover similar use cases. It uses OpenCL to accelerate image operations and OpenGL to render results. There is no scripting or UI and it is not very rapid prototyping friendly in the current form since it targets usage by C++ programmers only.

In conclusion, all of the listed graph-based tools are based on ITK and VTK. Other common dependencies are* Qt* [40],* Python* [41], and* boost* [42]. These libraries are very large and can be a hassle to build or to get working properly. In contrast, our package only depends on a single external library, SDL, which is small and easy to build on many operating systems. We have tested our package on Windows and Linux, and as soon as a working OpenGL 4.5 driver for macOS is available it will be supported as well. Therefore, we expect that in comparison to similar packages our solution will be the easiest to deploy, as long as the system's graphics driver is able to support at least OpenGL 4.5.

### 3.3. Usage Examples

**Figure 2:**
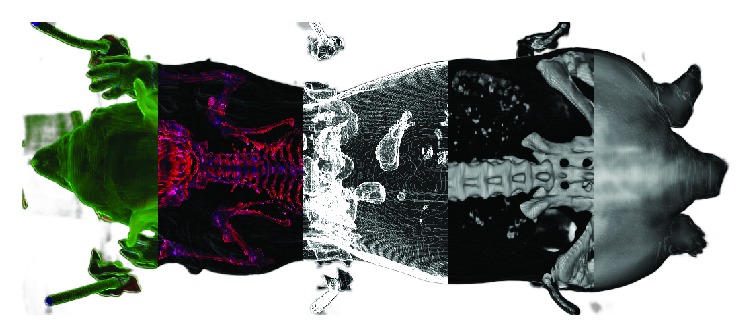
Selection of different renderings of CT data produced by the included volume renderer node and some supporting nodes. From left to right: solid with curvature as color; solid rescaled with curvature as color; edge/step function; solid rescaled; translucent.

The test system used for our benchmarking efforts was a consumer notebook with an Intel Core i7-4790S CPU @ 3.2 GHz (4 cores, 8 threads) and a NVIDIA GeForce GTX 965M graphics card with 4 GB memory, operating under Windows 8.1. The software was compiled using Visual Studio 2015 Update 3. All pipelines shown below are included in the release package as examples. We do not include MRI source data due to data protection. Good sources for initial test data are the* Digimouse* [43] and the *V*^∧^*3* [44] datasets.  Figure 2 is an example for some of the rendering modes possible with the built-in volume renderer node.

### 3.4. Distance Transform

**Figure 3:**
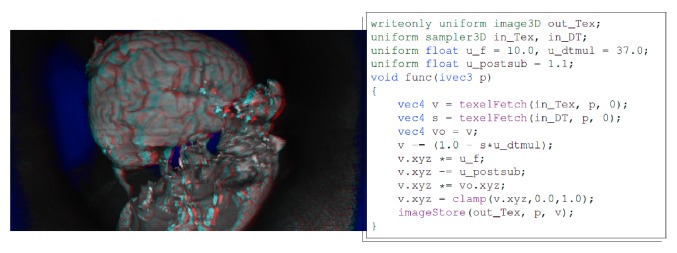
The distance transform from Table 1 combined with a custom compute node running the GLSL snippet on the right is a simple way to remove most of the skull in a head MRI scan. The result is red/cyan stereo-rendered.

We have performed a benchmark test, comparing the performance of a 3D distance transform implemented in multithreaded C++ and GLSL, respectively. Specifically, we chose the fast distance transform method from [45] because the algorithm is not a pointwise operation (and therefore not trivially GPU-parallelizable) and needs an initial preparation pass plus one pass for each axis (4 in total). The distance transform can be used for further processing. An example is given in Figure 3.

Given the result in Table 1, we conclude that our GPU variant is about two orders of magnitude faster than the CPU-based 3D distance transform for this specific computation, enabling almost interactive operation (e.g., changing the threshold and observing results). Figure 3 is an example for an extended use case: a custom compute node executes a snippet of GLSL code, which utilizes the distance transform algorithm to remove most of a human skull. The entire snippet runs in less than 4 ms for a 256 × 256 × 176 volume. While the rendered result is not perfect and a bit noisy, the approach was developed within few minutes on the fly, including the parameters used for the transformation. Excluding the distance transform (which has to be computed only once) but including volume rendering of the result in stereo 3D, the pipeline takes less than 20 ms to execute.

### 3.5. Segmentation

**Figure 4:**
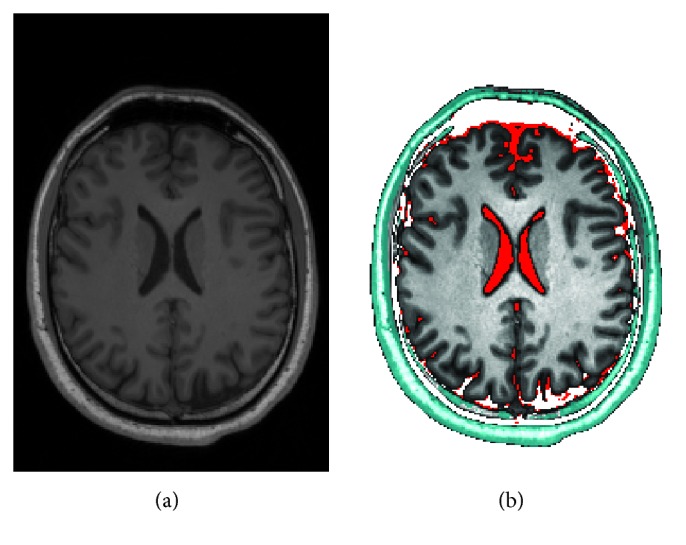
Slice of a brain MRI scan. (a) is normalized but otherwise unprocessed. (b) is segmented into skull/skin (teal), cerebrospinal fluid (red), and brain (grey) tissue. The brain tissue is contrast-enhanced.

A typical operation for medical image analysis is tissue segmentation. We have developed a simple proof-of-concept segmentation pipeline that extracts a specific intensity range followed by a smoothing and threshold operation to form a mask. This mask is then used to segment skin and skull from the remaining tissue. Another quickly developed code snippet performs the remaining segmentation and contrast enhancement. For visualization, a slice is extracted (Figure 4). Note that the segmentation was performed on the complete 256 × 256 × 176 volume instead of a single slice only. The whole pipeline executes in about 161 ms for this volume, of which the final segmentation snippet took 4 ms. An alternative pipeline that processes the same volume in 22 ms is also included in the examples.

### 3.6. Other Benchmarks

**Figure 5:**
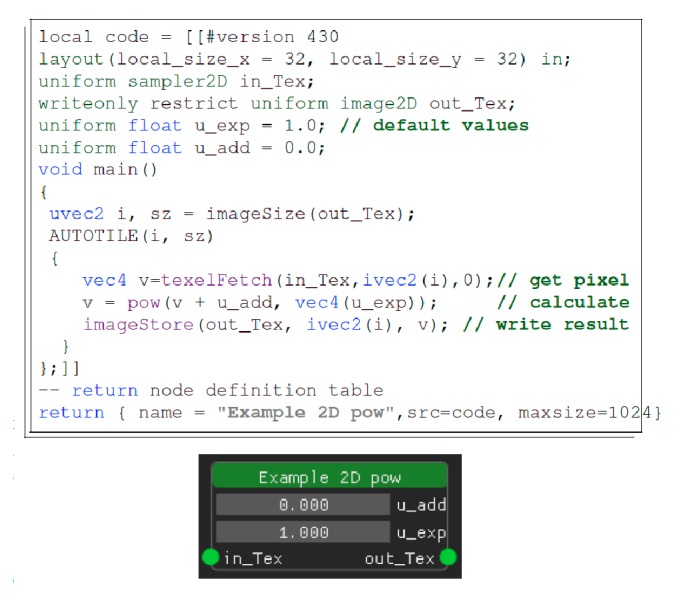
A minimal working example. This node calculates (*v* + add)^exp^ for every pixel and color channel in a 2D image, resulting in contrast enhancement. The only Lua code is the definition table in the last line; the rest is GLSL embedded in a Lua string. Missing interface functions are automatically induced. The resulting graphical representation is displayed in the small box/node (dark background, green title bar). More information can be found in the supplement (available here).

**Figure 6:**
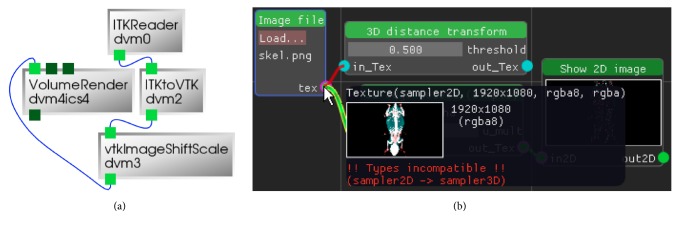
Comparison of our graph editor (b) with the interface of DeVIDE (a). Our graph editor shows a lot more detail, including a preview when hovering in-/outputs if possible. It also checks for misuse, that is, ensures connector type compatibility and prevents cycles. The depicted DeVIDE graph did not work, despite trying multiple variants. There was no sign of error when building the graph.

**Figure 7:**
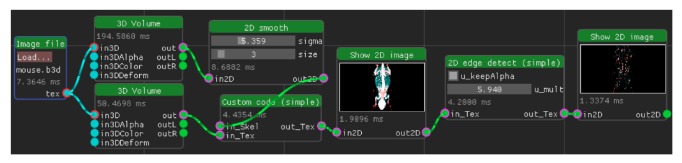
A dataflow graph to visualize a mouse CT volume. Both “3D Volume” nodes render a volume texture into a 2D image, given individual settings. In this example, the resulting 2D image is set to 4K resolution (3840 × 2160). An estimate of the time in milliseconds that a node spent computing on the GPU is displayed on each node, respectively. Any parameter/image changes propagate downstream, so that the whole graph updates itself as necessary.

We have included two more examples for point-wise operations into the benchmarks in Table 1: median filtering and intensity rescaling. Intensity rescaling is a two-step operation. First, minimal and maximal intensity value are determined and then each pixel value is scaled accordingly so that the output values are in [0 ⋯ 1]. Simple point-wise operations like this can benefit even more from GPU acceleration, such as Gaussian smoothing, arbitrary convolutions, edge detection, thresholding, and filtering. This kind of relatively simple operation can finish in a few milliseconds (ms) for typical input sizes (e.g., a volume of size 512^3^ voxels), allowing fully interactive use and parameter adjustment in real-time (Figures 4 and 5). Median filtering on the GPU is costly due to the sorting involved; sorting is implemented using a parallel sorting network [46].

## 4. Discussion

We expect that our framework will make GPU-accelerated data processing more accessible. While the examples given so far mainly target use cases in the medical domain, our method is not limited to these and can be used for many kinds of 3D data processing that involve images or parallelizable operations on a block of data. The motivation to create a self-contained framework for rapid prototyping and built-in visualization came from difficulties when developing certain methods to operate on CT/MRI data. The intended algorithms were too costly to execute on CPUs in any reasonable time, so GPU acceleration was a necessity. Many time-consuming problems during development could have been avoided if not only the inspection of data had been supported visually, but also the in-memory representation had been easily accessible. Our prototype of the pipeline was hardcoded in C++, using plain OpenGL [47], without rapid prototyping functionality or automatic memory management, thus changing parameters or rewiring pipeline connections required recompiling, and rerunning the whole program, often causing problems due to technical oversights and remaining bugs. Our current framework attempts to solve these problems. It accelerates the once time-consuming part of pipeline development, so that the implementation cycles for new features are much shorter and also much more efficient. Regarding the implementation, we intentionally rely solely on OpenGL for GPU acceleration, because it is the most compatible, complete, and platform-independent graphics and compute API currently available. Other options either are vendor-locked (CUDA), have no graphics capabilities (OpenCL), or are exclusive to an operating system family (DirectX, Metal). OpenGL does not come without problems however. GPU drivers are mostly proprietary, each implementing a different interpretation of the OpenGL specification, sometimes exposing implementation differences and bugs [4, 47]. This may affect the core implementation; therefore care has been taken to adhere to the OpenGL 4.5 specification, but some drivers may still cause problems. Practically, this may also cause user-written GLSL shaders to work fine on a specific setup but fail to compile or misbehave on another, if the respective driver's GLSL compilers exhibit differences.

We also chose deliberately to not only enable but also enforce all node computations to be performed by the GPU. While this rules out incorporating popular libraries such as ITK and OpenCV, we believe that this is the only way to ensure a consistently fast pipeline without unnecessarily slow legacy components. The current focus/use case of the application is novel development instead of reusing existing components in a new package. This is not expected to be a problem for most users, as this is what sets our framework apart from others.

Given the ability to type in code at run-time and inspecting results immediately, intuition-guided exploration of data is much easier and more interactive in comparison to existing solutions, as they offer mostly premade building blocks. The combination of rapid prototyping throughout our entire implementation, while at the same time also GPU-accelerating all calculations, allows a user to process larger datasets faster than with any of the other tools, as these were apparently not built with this kind of dynamism in mind.

A selection of algorithms is included in the first public release. They might not be sufficient to cover all use cases; therefore the use for medical research is not yet the primary focus of our package. However, even at this early stage of development, our software is useful for teaching and extension for specific tasks by anyone.

### 4.1. Future Work

In its current state the software is a stand-alone application rather than a library. Future functionality may include a program exporter to design entire processing pipelines inside the user interface and then export a single script that implements this dataflow graph. This would be useful for batch processing and inclusion in existing pipelines and is expected to greatly simplify deployment for end users. We also plan to include support for point clouds and regular 3D meshes, as they are strongly tied to graphics development and benefit greatly from GPU-accelerated processing. We believe combining the ability to manipulate and visualize these types of data in one package will be beneficial to other related fields. Moving away from OpenGL is not a plan for the immediate future but a long-term goal. Switching to Vulkan [48] as a backend would not only enable multithreaded, multi-GPU computation but also enable support for mobile devices (e.g., Android-based tablets) and hopefully minimize driver-specific behavior when compared to OpenGL.

## Figures and Tables

**Table 1:** Timings for selected operations on 3 different volumes. The speedup factor between CPU and GPU is calculated as the lowest CPU time (8 threads) divided by the GPU time. The 3D Euclidian distance transform operates on normalized (pixel values in [0 ⋯ 1]) volumes with a solidity threshold of 0.5. The CPU implementation is our own, including the multithreading. Intensity rescaling and median filtering use ITK's CPU-based implementation. The median filter takes the direct neighborhood of each voxel into account, that is, a box of 27 voxels in total.

Volume	CPU (1 thread)	CPU (8 threads)	GPU	Speedup 1 versus 8 threads	Speedup CPU versus GPU
Distance transform					
256 × 256 × 176	134.5 s	45.9 s	630 ms	2.9x	73x
512 × 512 × 19	63.2 s	20.3 s	217 ms	3.1x	93x
380 × 992 × 208	1631 s	627 s	4900 ms	2.6x	128x

Rescale intensity to [0 ⋯ 1]					
256 × 256 × 176	88 ms	68 ms	10.1 ms	1.29x	6.7x
512 × 512 × 19	38 ms	30 ms	7.2 ms	1.27x	4.17x
380 × 992 × 208	603 ms	457 ms	97.5 ms	1.31x	4.7x

Median filter					
256 × 256 × 176	4155 ms	1544 ms	97 ms	2.7x	15.9x
512 × 512 × 19	2120 ms	671 ms	48 ms	3.15x	14x
380 × 992 × 208	14 s	4800 ms	715 ms	2.9x	6.7x

## Data Availability

Program and source code are accessible at https://bitbucket.org/maxmalek/xcv. The mouse volume used in some of the examples was converted from the* Digimouse* dataset [43]. The human head MRI data set was donated under the condition of anonymity and cannot be published.

## Supplementary Materials

Supplementary Materials The supplement is a quick-start and developer's guide for our software, xcv. It describes how to extend the program, related APIs, and good GLSL practices to maximize compatibility across graphics drivers. The supplement also provides a hands-on example for the implementation of a new node type.

## References

[1] Fortmeier D. (2016). *Direct volume rendering methods for needle insertion simulation [Ph.D. Dissertation]*.

[2] Zhou J., Wang X., Cui H. (2016). Topology-aware illumination design for volume rendering. *BMC Bioinformatics*.

[3] Srinivasan R., Fang S. (2000). Integrating volume morphing and visualization. *Computational Geometry*.

[4] Smistad E., Bozorgi M., Lindseth F. (2015). FAST: framework for heterogeneous medical image computing and visualization. *International Journal for Computer Assisted Radiology and Surgery*.

[5] Akhloufi M. A., Gariepy F., Champagne G., Owens J. D., Lin I., Zhang Y., Beretta G. B. GPGPU real-time texture analysis framework.

[6] Broussard R. P., Ives R. W., Owens J. D., Lin I., Zhang Y., Beretta G. B. Using a commercial graphical processing unit and the CUDA programming language to accelerate scientific image processing applications.

[7] Eklund A., Andersson M., Knutsson H. (2012). fMRI analysis on the GPU—Possibilities and challenges. *Computer Methods and Programs in Biomedicine*.

[8] Dachille F., Kreeger K., Chen B., Bitter I., Kaufman A. E., Kaufman A. E., Straßer W., Knittel G., Pfister H., Spencer S. N. High-quality volume rendering using texture mapping hardware.

[9] Hanwell M. D., Martin K. M., Chaudhary A., Avila L. S. (2015). The Visualization Toolkit (VTK): Rewriting the rendering code for modern graphics cards. *SoftwareX*.

[10] Sanders J., Kandrot E. (2011). *CUDA by example: an introduction to general purpose GPU programming*.

[11] Yang R., Welch G. (2002). Fast image segmentation and smoothing using commodity graphics hardware. *Journal of Graphics (GPU & Game) Tools*.

[12] Igual F. D., Mayo R., Hartley T. D. R., Çatalyürek Ü. V., Ruiz A., Ujaldon M., Chapman B. M., Desprez F., Joubert G. R., Lichnewsky A., Peters F. J., Priol T. Exploring the GPU for enhancing parallelism on color and texture analysis.

[13] (2017). *K. O. W. Group, The opencl specification, version 2.2*.

[14] Cozzi P., Riccio C. (2012). *OpenGL Insights*.

[15] Bailey M., Cunningham S. Computer graphics shaders using OpenGL 4.X.

[16] Bailey M., Cunningham S. (2011). *Graphics Shaders: Theory and Practice*.

[17] Jeremias P., Quilez I. Shadertoy: Learn to create everything in a fragment shader.

[18] Reunanen M. (2017). *Times of change in the demoscene: A creative community and its relationship with technology [Ph.D. dissertation]*.

[19] Hartmann D. *Digital Art Natives: Praktiken, Artefakte und Strukturen der Computer-Demoszene*.

[20] Marin-Vega H., Alor-Hernández G., Zatarain-Cabada R., Barron-Estrada M. L., García-Alcaraz J. L. (2017). A Brief Review of Game Engines for Educational and Serious Games Development. *Journal of Information Technology Research*.

[21] Turinsky A. L., Fanea E., Trinh Q. (2008). CAVEman: Standardized anatomical context for biomedical data mapping. *Anatomical Sciences Education*.

[22] Ierusalimschy R., de Figueiredo L. H., Filho W. C. (1996). Lua-an extensible extension language. *Software: Practice and Experience*.

[23] Lantinga S. (2017). *Simple DirectMedia Layer*.

[24] Cornut O. https://github.com/ocornut/imgui/.

[25] Group K. O. W. *The opengl graphics system: A specification (version 4.5 (core profile)*.

[26] Everitt C., Sellers G., McDonald J., Foley T. https://www.slideshare.net/CassEveritt/approaching-zero-driver-overhead.

[27] Collet Y. (2015). *Zstandard*.

[28] Johnson H. J., McCormick M., Ibáñez L., Consortium T. I. S. *The ITK Software Guide*.

[29] The HDF Group *(1997-2017) Hierarchical data format, version 5*.

[30] https://itk.org/Wiki/ITK/File_Formats

[31] Barrett S. https://github.com/nothings/stb.

[32] Ritter F., Boskamp T., Homeyer A. (2011). Medical image analysis. *IEEE Pulse*.

[33] Botha C. P. (2004). DeVIDE: The delft visualisation and image processing development environment. https://graphics.tudelft.nl/Publications-new/2004/BO04a.

[34] Gabr R. E., Tefera G. B., Allen W. J., Pednekar A. S., Narayana P. A. (2017). Erratum to: GRAPE: a graphical pipeline environment for image analysis in adaptive magnetic resonance imaging. *International Journal for Computer Assisted Radiology and Surgery*.

[35] Szalo A. E., Zehner A., Palm C. (2015). GraphMIC. *Bildverarbeitung für die Medizin 2015*.

[36] Schroeder W., Martin K., Lorensen B. (2004). *The Visualization Toolkit*.

[37] Wolf I., Vetter M., Wegner I. (2005). The medical imaging interaction toolkit. *Medical Image Analysis*.

[38] Nolden M., Zelzer S., Seitel A. (2013). The medical imaging interaction toolkit: Challenges and advances: 10 years of open-source development. *International Journal for Computer Assisted Radiology and Surgery*.

[39] Jeong W.-K., Pfister H., Fatica M. (2011). Medical image processing using GPU-accelerated ITK image filters. *GPU Computing Gems Emerald Edition*.

[40] Blanchette J., Summerfield M. (2006). *C++ GUI Programming with Qt 4*.

[41] van Rossum G., Chase J., Seshan S. Python programming language. https://www.usenix.org/publications/proceedings/?f[0]=im_group_audience%3A114.

[42] Polukhin A. (2017). *Boost C++ Application Development Cookbook*.

[43] Dogdas B., Stout D., Chatziioannou A. F., Leahy R. M. (2007). Digimouse: a 3D whole body mouse atlas from CT and cryosection data. *Physics in Medicine and Biology*.

[44] Roettger S. http://lgdv.cs.fau.de/External/vollib/.

[45] Felzenszwalb P. F., Huttenlocher D. P. (2012). Distance transforms of sampled functions. *Theory of Computing. An Open Access Journal*.

[46] Batcher K. E. Sorting networks and their applications.

[47] Barczak J. (2014). *OpenGL Is Broken. The Burning Basis Vector [blog]*.

[48] Group T. K. V. W. (2017). *Vulkan 1.0.66 - a specification*.

