# Supplementary material for: Instant Feedback Rapid Prototyping for GPU-Accelerated Computation, Manipulation, and Visualization of Multidimensional Data
Source: Int J Biomed Imaging. 2018 Jun 3;2018:2046269. doi: 10.1155/2018/2046269 (PMC6008673; doi:10.1155/2018/2046269)
Supplement: Supplementary Materials — The supplement is a quick-start and developer's guide for our software, xcv. It describes how to extend the program, related APIs, and good GLSL practices to maximize compatibility across graphics drivers. The supplement also provides a hands-on example for the implementation of a new node type. [file 2046269.f1.pdf]

Xcv extension guide

Maximilian Malek, Christoph W. Sensen

Contents

1 Introduction **1**

2 Extension via nodes, using the Lua API **1**
2.1 Dataflow graph 1
2.2 Node API 2
2.3 User Interface 2

3 Good practices for GLSL **3**

4 Extension via plugin, using the C++ API **3**

A Appendix **4**
A.1 Recognized definition table entries 4
A.2 Data format string 5
A.3 Swizzle mask string 5
A.4 Texture flags string 5
A.5 Buffer flags string 6

B Hands-on Example **6**
B.1 Node: Noise field 6

1 Introduction

This document provides an overview of possibilities to extend our real-time rapid-prototyping framework **xcv**. The most up-to-date documentation and source code can be found at https://bitbucket.org/maxmalek/xcv.

There are two ways to add extra functionality: Lua [1] scripting and C++ plugins.

The first part of this document describes the more user-friendly Lua API. The second part explains the C++ extension API and plugin creation.

2 Extension via nodes, using the Lua API

Xcv performs data manipulation and most heavy computations via GLSL compute shaders. Lua 5.3 is used as a glue language for user interface (UI), light computation, automatic resource management and orchestration of internal components. GLSL shader code is typically embedded as a Lua string or generated on the fly and further compiled into a *shader program*, and then invoked as needed. Most operations can (and should) be performed using the Lua API, such as reading and writing data (file formats are detected automatically where applicable, *e.g.* image files), interacting with the GPU (textures, buffers, shaders, etc.), user interaction, automation, etc. The rendering process is scripted as well but is outside of the scope of this document.

Resource management is integrated with the Lua garbage collector and handled automatically.

2.1 Dataflow graph

A *node* has defined inputs and outputs that may perform operations on the data passing through, but is not allowed to have global side effects. Data that pass through the graph are immutable, *i.e.* nodes never change the data directly. This ensures that the same input can be passed to multiple nodes without risking unexpected changes to the data before all nodes are done processing. This has to be taken care of when implementing a node – make sure not to change the input data. A node typically creates its own output data, which is then passed to nodes down-stream.

```lua
local glslcode="..."--the shader
local function init(node)
    -- init node
end
local function recalc(node, inp)
    -- do something with inputs
end
return { -- definition table
    init = init,
    recalc = recalc,
    src = glslcode,
}
```

Figure 1: Example node definition table and some interface functions.

2.2 Node API

A node is a single Lua script that exposes functions called by the core scripts. The script file should be self-contained, i.e. not rely on 3rd-party libraries or anything beyond the core scripts. A node script should not write to the global Lua namespace, such as register global functions or variables. In order to prevent accidental access to globals, node scripts are sandboxed: They can see the same global namespace as the rest of the application, but changes to the global namespace are restricted and contained within the node script to some degree. This feature exists solely to catch implementation errors, but is not a "safe" sandboxing method to contain *e.g.* malicious scripts.

A node script typically ends by returning its *definition table* (Figure 1), containing functions and data definitions. All entries in the definition table are optional. Recognized table entries are listed in Subsection A.1. All node *interface functions* get passed one or more parameters when called by the core scripts. The first parameter is always the respective node itself.

For nodes with a simple compute shader with "obvious" inputs and outputs but some missing interface functions, the core scripts will look at the definition table and try to provide the missing functions automatically. If this is not possible and the node does not satisfy all requirements, it will fail to load and an error is displayed.

The automatisms are as follows:

- If **src** is specified, compile the shader code into `node.shader`
- If **file** is specified, load shader code from that file and use it as if it was *src*
- Inspect the shader and deduce connectors for the node.
  - Inputs start with **in_**
  - Outputs start with **out_**
  - Tunables/knobs start with **u_**
- If the shader has *buffers* as input or output, require user implemented `recalc()` / `makeOutput()` functions.
- If the shader has a single *image* as output:

➔ Allocate output image based on input, in specified format and size (Subsection A.1) if supplied, otherwise same as input:
  - If single input, use that
  - If multiple inputs: use *outGen* definition (Subsection A.1)
- Run compute shader with inputs and outputs
- Return generated output

```lua
local function drawUI(node)
    if imgui.Button(" + ") then node.x = node.x+1 end
    imgui.SameLine()
    if imgui.Button(" - ") then node.x = node.x-1 end
    imgui.Text("x: " .. node.x .. ", y: " ..node.y)
end
local function drawDetailUI(node)
    drawUI(node) -- repeat node UI
    imgui.Separator()
    local changed, newy =
        imgui.SliderInt("Param y", node.y, 0, 100)
    if changed then
        node.y = newy
        -- update and propagate downstream
        node:onSomethingChanged()
    end
end
```

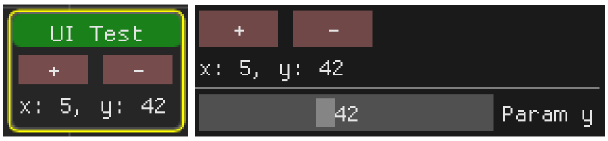

Figure 2: UI example with code. The left image is the node as it is drawn in the graph editor, the right image is how the node appears in the detail panel when selected.

Whenever a node changes and must re-evaluate its inputs, `node:onSomethingChanged()` must be called to propagate changes through the graph. Dependant nodes' `recalc()` method will be automatically invoked if required.

If a node script requires functions from a plugin, use `require "libname"` in its `init()` function.

2.3 User Interface

The user interface is exclusively built with *dear imgui* [2]. In contrast to other ("typical"/retained-mode) UIs, logic happens while widgets are drawn ("immediate mode gui"). A widget is simply a function (Figure 3). There is no state or UI setup phase, everything is drawn on demand and thus recreated in every frame. This makes it very easy to modify the UI in flight. Figure 2 is a more complete example with graphical representation.

```lua
function drawUI()
    if imgui.Button("Press") then
        -- button was pressed
    end
end
```

Figure 3: Immediate Mode UI

Since nodes are implemented in Lua, a good place to look at currently exported functionality is src/guibase/lua_imgui.cpp – this file contains the interesting bits and implementation of the imgui Lua bindings.

The file bin/luacore/imgui.lua contains more useful functions implemented on top of the C++/Lua interface.

Refer to the *dear imgui* repo at https://github.com/ocornut/imgui/ for further examples and more documentation.

3 Good practices for GLSL

Compute shaders are exclusively implemented in GLSL and compiled by the graphics driver. Before shader code is passed to the graphics driver, some modifications are done to enhance compatibility with drivers, conditionally enable features based on hardware capabilities, and to provide macros for easier use. The most important macro is `AUTOTILE(index, size)` that implements automatic tiling of large input data. Automatic tiling ensures that the graphics driver will not reset the GPU for shaders that would otherwise take too long to execute. For each kernel invocation, `AUTOTILE(index, size)` generates a suitable *work index*, for all *size* pixels/elements. Both *index* and *size* must have the same type and can be vector types. Note that the **maxsize** parameter must be set in the node definition, otherwise autotiling is disabled. See *bin/shaders/comp.inc* for the implementation.

```
#version 430
layout(local_size_x=32, local_size_y=32) in;
uniform sampler2D in_Tex;
writeonly restrict uniform image2D out_Tex;
uniform float u_exp = 1.0, u_add = 0.0;
void main()
{
 uvec2 i, sz = imageSize(out_Tex);
 AUTOTILE(i, sz)
 {
    vec4 v = texelFetch(in_Tex,ivec2(i),0);
    v = pow(v + u_add, vec4(u_exp));
    imageStore(out_Tex, ivec2(i), v);
  }
};
```

Figure 4: A simple GLSL compute shader.

Naming conventions: Uniforms should be prefixed by **in_**, **out_**, **u_**, respectively to make them appear in the default UI (refer to Subsection 2.1). Add the `readonly` and `writeonly` keywords to inputs and outputs where applicable to catch misuse, and add the `restrict` keyword where appropriate – usually outputs can be safely marked as `restrict`. Preferably use *sampler* uniforms instead of *image* uniforms for reading data, and `texture()`, `textureLod()` or `texelFetch()` instead of `imageLoad()` [1]. Do not add `layout()` qualifiers to uniforms unless necessary – the framework handles internal OpenGL details like binding points and uniform locations automatically if they are not specified. Note that the GLSL compiler optimizes away unused uniforms – they will not appear in the UI if they are not used. Refer to Figure 4 for a small GLSL shader that follows the guidelines outlined in this section.

4 Extension via plugin, using the C++ API

Plugins are implemented as dynamic libraries and can be used to extend the core with functionality that would be too complicated or too slow to be implemented in Lua. Its intended purpose is the extension of image format support and the addition of extra Lua functions by exporting a table of functions that is subsequently loaded by the core.

Figure 5 shows a simplified plugin skeleton. API versioning and implementation details are automatically handled by the `MAKE_PLUGIN_*` and `*_FillHeader` macros. Compared to the Lua API, the C++ API is more limited and has no direct access to GPU functionality. The `MAKE_PLUGIN_IMPORT` macro may be used to retrieve a list of function pointers supplied by the core, which comprises functions for error reporting, image format conversion, and interaction with the embedded Lua interpreter.

The number of available functions is currently very limited and will be extended in future. The current main use case is saving images, optionally converting them to a desired format if required by the target file format.

Refer to the included *stb_image* plugin for a comprehensive example – it implements both loading and saving images and also interacts with the core API.

```
#include <stdlib.h>
#include "api_plugin.h"
// Load functions provided by core
// Use only if needed.
//MAKE_PLUGIN_IMPORT(api);
static const PluginAPIFuncs funcs =
{
  PluginAPIFuncs_FillHeader,
  freeImageData, // User-defined.
  loadImageFile, // See api_plugin.h
  saveImageFile, // for prototypes.
  getImageFormatsAvailLoad,
  getImageFormatsAvailSave,
};
static const PluginAPIDef plugin =
{
  PluginAPIDef_FillHeader,
  NULL, // Not needed here
  &funcs
};
// Make visible to core
MAKE_PLUGIN_EXPORT(plugin)
```

Figure 5: Plugin skeleton. C++ code.

To compile a plugin, make sure the C++ headers in *src/api/*.h* are visible to the compiler. No other headers or libraries are required aside from the C standard library.

[1] This increases compatibility with OpenGL drivers, as `imageLoad()` requires a `layout(<format>)` qualifier that may or may not cause problems when the actual internal format of the texture is not what the shader expects. Apparently this is *not* required for `imageStore()`, so it is best to leave the format qualifier away altogether and use samplers, which have no such restriction.

A Appendix

A.1 Recognized definition table entries

- **name**: Human-readable name of the node. Appears in its title text area.
- **category**: Used as a prefix before the name in the "new node" menu. (Typically something like "compute", "render", "input", "output", but anything can be used)
- **desc**: Longer description. Shown in the info panel.
- **tags**: Space-separated list of words that will be used by the quick node search function. Words from *name*, *desc*, *category* and *author* are already part of the search, but *tags* may be used to include additional keywords to improve searchability.
- **author**: Shown in the info panel.
- **references**: Single string or table of strings. Treated as an URL and is made clickable in the UI.
- **src**: GLSL source code
- **file**: File name to load GLSL source from
- ***init(node)***: Function that is called whenever a node is initialized.
  - Expected to set default values
  - Called when code is reloaded / user presses F5
  - Possibly called multiple times throughout a node's lifetime
- ***makeOutput(node, inputs, name)***: Function that must return a new object for a given output variable *name*.
  - Automatically inferred when not present
  - *inputs* is a table, indexed by the name of the input connector, and its associated input object (e.g. texture, buffer, etc)
  - Set to *false* if outputs are to be constructed in recalc() (Saves memory for custom recalc() implementations)
- ***recalc(node, inputs)***: Function that is expected to fill/return output objects (*i.e.* that invokes the compute shader)
  - *inputs* is same as for makeOutput()
  - Automatically inferred when not present
  - There are 3 ways to fill outputs:
    1. The function itself adds entries to `node.RESULTS[cname] = x`, where *cname* is the name of the output connector. Nothing is returned from the function.
    2. If the node has a single output connector: `return resultObj`. The name of the connector is inferred.
    3. Return a table with results, e.g. `{outTex1=x, outTex2=y, outBuf=z}` to populate connectors *outTex1*, *outTex2*, *outBuf*.
- ***serialize(node)***: Function called when serializing node state. Can return a table, string or number describing the node state. The returned value must be serializable.
- ***deserialize(node, t)***: Function called when node state is to be restored, if this node has previously returned something from serialize(). *t* contains the value originally returned from serialize().
- ***drawUI(node)***: Called when a node is drawn.
  - Whatever is drawn in this function is drawn directly on the node in the graph. The node is resized to fit.
  - Automatically inferred when not present (default: draw widgets for uniforms prefixed with **u_**).
  - If a true value is returned, `node:onSomethingChanged()` is called.
- ***drawDetailUI(node)***: Called when a node is focused. Defines the detail panel content of the node.
  - Whatever is drawn in this function is drawn into the detail panel when the node is focused.
  - Automatically inferred when not present (default: draw outputs, if present).
  - If a true value is returned, `node:onSomethingChanged()` is called.
- ***drawOutput(node, name, obj)***: Called when drawing output *name* (usually into the detail panel).
  - This function is useful to specialize the way an output is drawn.
  - *obj* is the associated output object.
  - Call `drawOutputDefault(node, name, obj)` to forward drawing to the default handler, if no specialization required for a given *obj*.
- ***openContextMenu(node)***: Called when the user right-clicks the node, just before the context menu is opened.
  - This function is intended to prepare context menu entries before drawing.
- ***drawContextMenu(node)***: Called when a node context menu is to be drawn.
  - If this function is present, the node gets a blue border to indicate that there is an extra context menu.
- ***update(node, dt)***: Called every frame.
  - *dt*: Time difference to the prev. frame, in seconds.

  – Useful to run simulations or apply changes to a node as time passes.
- **outGen**: Table that specifies how outputs are to be generated, if generating output automatically. Format: `{outputName = X, ...}` where X can be:
  – a string, *e.g.* `outTex = "inTex2"`. This is equivalent to `outTex = {input = "inTex2"}`.
  – a table, *e.g.* `outTex = {`
    `input = "inTex2",` – This specifies that properties of inTex2 should be used to create outTex.
    `format = "rgba16f",` – If present, overrides the input data format, see Subsection A.2.
    `swizzle = "rgba",` – If present, override the swizzle mask, see Subsection A.3.
    `flags = "lmiB",` – If present, override the texture flags, see Subsection A.4.
  `}`
- **extraInputs**: A table that allows to specify extra input connectors that are not part of the shader or not picked up automatically. Specifies a connector and its GLSL type.
  Like this: `{inputTex = glsl.sampler3D, inputBuf = glsl.buffer}`.
  Input objects are accessible in the *inputs* table passed to `recalc()` and `makeOutput()`.
- **extraOutputs**: Like *extraInputs*, but for output connectors.
- **maxsize**: Maximal allowed input size for one shader dispatch. Required for automatic tiling (*i.e.* if this is omitted, automatic tiling is disabled even if the `AUTOTILE` macro is used!). Can be a number or a table `{x,y,z}` with different size limits for each dimension.

A.2 Data format string

The data format string specifies the number of color channels and underlying value type of a texture.

r, rg, rgb, rgba specify 1-4 channels, respectively.
8, 16, 32 specifies the number of bits per channel
i, ui, f suffix specifies integer, unsigned integer, or floating-point data.

Not all combinations are valid (e.g. 8-bit float). Always specified as *channels*, *bits*, *suffix*.
Examples:
rgba8ui: 4 channels with 8 bits per color (typical for color images with transparency)
rg16f: 2 channels of 16 bit floats (half precision)
r16ui: 1 channel of 16 bit unsigned ints (typical for MRT/CT scans)
rgb32f: 3 channels of 32 bit floats (typical for HDR color images)

A.3 Swizzle mask string

A valid swizzle mask contains exactly 4 characters out of **{rgba01}**. It specifies the how a pixel is sampled from a texture. *rgba* stands for channels 1-4 respectively, 0 returns a zero value, 1 returns the maximal value.
Examples:
rgba: Default: 3 colors with alpha
rrr1: Greyscale, fully opaque
rggg: First channel is passed through, second channel for everything else

If not present or empty, the swizzle mask is automatically selected based on the number of channels:
1 → **rrr1** (greyscale)
2 → **rrrg** (luminance + alpha)
3 → **rgb1** (R/G/B colors without alpha)
4 → **rgba** (colors with alpha)

Any unrecognized characters cause an error.

A.4 Texture flags string

String that may contain the following characters:
l (lower-case L) : Texture sampling uses linear interpolation (default: nearest-neighbor interpolation)
m : Enables mipmapping. Uses more memory. Usually not required.
i : Use integral texture, for integer values. (default: normalized float)

B : Backup texture to system RAM after data upload. In case of a recoverable GPU crash, the texture is automatically restored. This is an internal flag that should normally not be used. The backup is NOT updated automatically when shaders write to the texture.

The default is an empty string. Any unrecognized characters are silently ignored.

A.5 Buffer flags string

String that may contain the following characters:

r: Buffer content can be memory-mapped for reading.
w: Buffer content can be memory-mapped for writing.
u: Buffer content can be updated after initial creation (via `buffer:uploadBytes()`)
p: Buffer mapping is persistent. Must be used with r or w. Data written by the GPU can be seen by the CPU and vice versa.
B: Backup buffer contents to system RAM after data upload. In case of a recoverable GPU crash, the buffer is automatically restored. This is an internal flag that should normally not be used. The backup is *not* updated automatically when shaders write to the buffer, or the buffer is changed by writing to mapped memory. If this flag is present, the backup *is* updated when buffer:uploadBytes() is called or the buffer is first created.

The default is an empty string. Any unrecognized characters are silently ignored.

B Hands-on Example

This section provides a hands-on example that explains the process of creating a node in detail. For better debugging support, enable *Debug/Developer mode* in the settings in the top panel. After enabling it, in order to make the change fully effctive, reload all scripts by pressing F5.

B.1 Node: Noise field

For this example we create a node that generates a 3D noise texture. This could be used for *e.g.* testing denoising algorithms or noise-tolerant surface reconstruction.

Since one thread fills one pixel, the shader is going to be fairly simple and we can start from the shader in Figure 4. There is a single output, no input, and no uniforms (for now). Since the output texture is 3D, it makes sense to process in 8x8x8 blocks (`layout(...) in` qualifier). This launches 512 threads per block, which is well within of OpenGL's limitation of 1024 threads per block. The main loop is adapted to use a $\text{noise}() : vec3 \rightarrow vec4$ function. This function will be filled in later.

```
#version 450
layout(local_size_x=8, local_size_y=8, local_size_z=8) in;
writeonly restrict uniform image3D out_Tex;

vec4 mynoise(vec3 uv) { ... }

void main()
{
    uvec3 i, sz = imageSize(out_Tex);
    AUTOTILE(i, sz)
    {
        vec3 uv = (vec3(i) + 0.5) / vec3(sz);
        vec4 f = mynoise(uv);
        imageStore(out_Tex, ivec3(i), f);
    }
};
```

Before the compute shader can be tested some surrounding code is required that transforms it into a node. This is done with the Lua code below.

```
local code = [[
<GLSL code here>
]]
local function makeOutput(node, name, inputs)
    --                          w    h    d    format   flags swizzle
    return gpu.texture.new(256,256,256, "r16f", "l", "rrr1")
end
return {
    makeOutput = makeOutput,
    src = code,
```

```
    maxsize = 512,
}
```

Note that as there is no input to derive the output from, the node has to generate its output explicitly by providing a `makeOutput()` function. The parameters can be ignored since there is only one output and no inputs. Multiple outputs would have to be distinguished by *name*. `recalc()` is not required in this case as the shader is simple enough so that the framework can infer the rest.

For the noise texture half-float (16-bit) precision should be enough, since the resulting values are usually in the range $[-1..1]$ and higher precision would waste memory. This may not be an issue with 2D images, but 3D images can take up a lot of space. The texture created in this example uses 32 MB RAM ($256^3 \times 1$ channels $\times$ 2 bytes per channel); with 32-bit floats this would be twice as much. In comparison, a `"rgba32f"` texture of the same size requires 256 MB RAM. The `"l"` flag enables linear interpolation.
An easy "noise" function for testing can be created using $sin()$:

```
vec4 noise(vec3 uv)
{
    vec4 q = vec4(uv*42., 0.);
    return vec4(0.33 * dot(sin(q), vec4(1)));
}
```

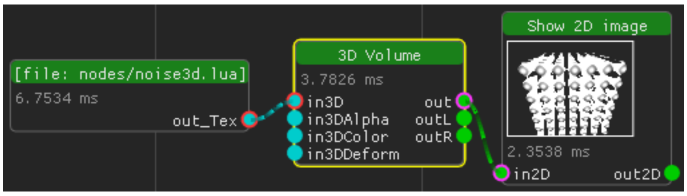

This function returns the same value on all four output channels, so it can be easily visualized using a volume renderer node. To make the result a bit more controllable, some multipliers can be added easily:

```
// ...at the top, add...
uniform float u_mul = 1.0, u_scale = 42.0;
// ...in noise(), change...
    vec4 q = vec4(uv * u_scale, 0.);
//... in main(), change...
        vec4 f = mynoise(uv) * u_mul;
```

Press F5 to reload the node. Suitable widgets for *u_mul* and *u_scale* show up directly on the node thanks to the **u_** prefix. Each uniform's value can now be changed easily with the mouse. Now that the "noise" is adjustable, the actual function can be improved, *e.g.* using the excellent 4D perlin noise [3] function from [4]. For later, add another uniform *u_time* as the 4th dimension.

```
uniform float u_time = 0.0;
// ...
float Perlin4D( vec4 P ) { ... }
vec4 mynoise(vec3 uv)
{
    vec4 q = vec4(uv * u_scale, u_time);
    return vec4(Perlin4D(q));
}
```

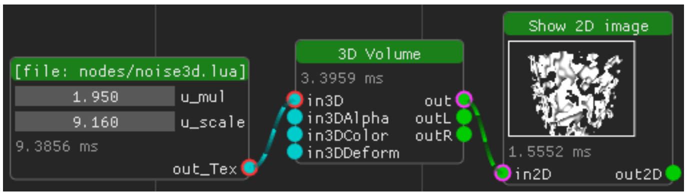

The new noise looks much more random and is smooth. In order to add one last feature, three new functions are required:

```
-- <previous Lua code here>
local function init(node)
    node.time = node.time or 0 -- default value
end
local function drawUI(node)
    node:drawUIDefault() -- draw the default as well
    local changed
    changed, node.anim = imgui.Checkbox("Animate",
        node.anim)
end
local function update(node, dt)
    if node.anim and node._shader then
        node.time = node.time + dt
        node._shader:setuniform("u_time", node.time)
        node:onSomethingChanged() -- recompute
    end
end
return {
    init = init,
    drawUI = drawUI,
    update = update,
    -- <other entries here>
}
```

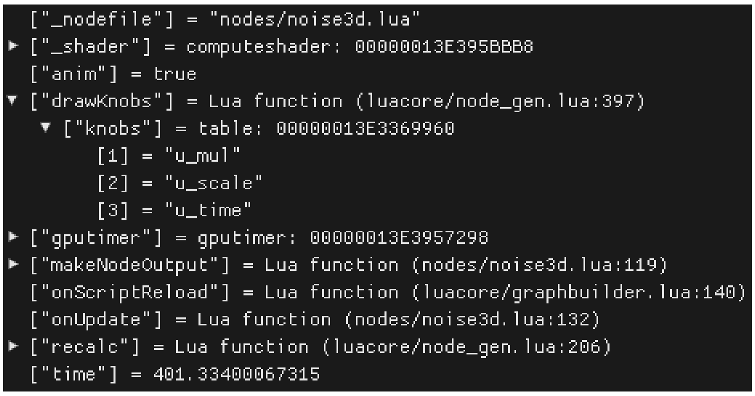

(a) Integrated object inspector

With this addition, the node gets a checkbox that, when enabled, causes the noise to change over time. Since the node has no `node.time` member at this point, `init()` is required to assign a default value (otherwise Lua would throw an error when attempting arithmetics with a *nil* value). By defining a custom `drawUI()` function, the "built-in" version gets overridden. The custom function asks the framework do its default and then adds a checkbox underneath. The `update()` function accumulates the elapsed time and updates the shader (and anything that depends on its output) as necessary.

In case there are problems during the implementation, a useful feature is the live object inspector that is available when developer mode is enabled. It displays all variables attached to an object and can recursively inspect an object hierarchy. Right-clicking a node brings up a context menu that contains an entry to open an object inspector for that node.

When the node is finished, a few more descriptive fields can be added to the definition table:

```
return {
    name = "3D Noise",
    category = "compute",
    desc = "Produces 3D noise of various flavors.",
    tags = "perlin",
    references = { "https://github.com/BrianSharpe/Wombat", "http://doi.acm.org/10.1145/325165.325247" },
    author = "John Doe",
    -- <previous entries follow...>
}
```

This concludes this example. Further extensions are implemented in the *Noise3D* node included in the software package, including serialization, more noise types, and multi-channel noise.

References

[1] R. Ierusalimschy, L. H. de Figueiredo, and W. C. Filho, "Lua-an extensible extension language," *Softw., Pract. Exper.*, vol. 26, no. 6, pp. 635–652, 1996. [Online]. Available: https://doi.org/10.1002/(SICI)1097-024X(199606)26:6<635::AID-SPE26>3.0.CO;2-P

[2] O. Cornut, 2017, (retrieved 2017-12-12). [Online]. Available: https://github.com/ocornut/imgui/

[3] K. Perlin, "An image synthesizer," in *Proceedings of the 12th Annual Conference on Computer Graphics and Interactive Techniques, SIGGRAPH 1985, San Francisco, California, USA, July 22-26, 1985*, P. Cole, R. Heilman, and B. A. Barsky, Eds. ACM, 1985, pp. 287–296. [Online]. Available: http://doi.acm.org/10.1145/325334.325247

[4] B. Sharpe. (2017) An efficient texture-free glsl procedural noise library. (retrieved 2018-01-12). [Online]. Available: https://github.com/BrianSharpe/Wombat/
